# Negative Tone Metallic Organic Resists with Improved Sensitivity for Plasma Etching: Implications for Silicon Nanostructure Fabrication and Photomask Production

**DOI:** 10.1021/acsanm.2c02986

**Published:** 2022-11-30

**Authors:** Ahmad Chaker, Hayden A. Alty, Paul Winpenny, George F. S. Whitehead, Grigore A. Timco, Scott M. Lewis, Richard E. P. Winpenny

**Affiliations:** † Department of Chemistry, The University of Manchester, Oxford Road, Manchester M13 9PL, U.K.; ‡ The Kavli Nanoscience Institute, California Institute of Technology, 1200 East California Boulevard, 107-81, Pasadena, California 91125, United States; § Sci-Tron Ltd, 34 High Street, Aldridge, Walsall WS9 8LZ, U.K.

**Keywords:** metal−organic resist, substitution, high selectivity, good sensitivity, electron beam lithography

## Abstract

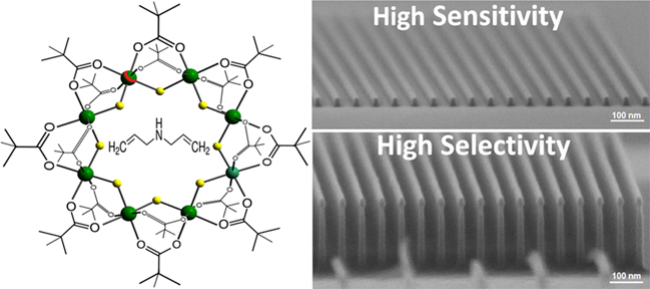

Metal–organic materials such as [NH_2_(CH_2_–CH=CH_2_)_2_][Cr_7_NiF_8_(Pivalate)_16_] can act as negative tone resists for electron beam lithography (EBL) with high-resolution patterning of sub-40 nanometer pitch while exhibiting ultrahigh dry etch selectivities >100:1 and giving line dose exposures >11,000 pC/cm. It is clear that the resist sensitivity is too low to be used to manufacture the latest nanoscale photomasks that are suitable for extreme ultraviolet lithography. Therefore, the focus of this work here is to improve the sensitivity of this resist while maintaining its resolution and dry etch selectivity. Using our latest Monte Carlo simulation called Excalibur, we predict that the sensitivity would increase by a factor of 1.4 when the nickel atom is substituted by a cadmium atom. EBL studies showed an excellent agreement with the simulation, and plasma etching studies demonstrated that this did not affect the dry etch performance of the resist which remains very good with a selectively of ca. 99:1 for the etching of silicon at these resolutions with a low sensitivity of 7995 pC/cm.

## 1 Introduction

Production of next generation dynamic random access memory now requires physical critical dimensions to be 17.5 nm half-pitch.^1^ This gives rise to significant challenges ahead to improve current lithography tools and materials to achieve this demand by the semiconductor industry.^2^ To achieve this target, 193 immersion lithography has been supplanted by the arrival of extreme ultraviolet lithography (EUVL) and this technique will require photomasks that are suitable for the method.^3−5^ For today’s EUVL photomask, the nanoscale feature sizes on the photomask are 40 nm half-pitch. Electron beam lithography (EBL) is the current technology that is able to produce high-resolution patterns that is required for these photomasks for EUVL.^6^ With each technology node, the features on the photomask get denser and denser, which will decrease the normalized image log slope from this elevated background dose with each generation of the photomask, making them tougher and tougher to produce because the time taken to fabricate these photomasks gets longer and longer, meaning that this is becoming a significant bottleneck.^7^ To alleviate this issue, one can either expose the resist using a lower acceleration voltage or design new electron beam resists that are more sensitive to the electron beam.^8^ Lowering the energy of electrons will broaden the electron beam leading to larger feature sizes. Therefore, the only option is to design new resist materials to meet this demand.^9^

Traditional resists for EBL are based on organic polymers, such as polymethyl methacrylate (PMMA) or ZEP520A.^10,11^ These two resists are widely used as positive tone resists; however, several studies show the ability to use those resists for a negative tone.^12,13^ Duan et al. reported that 8 nm lines with a pitch of 24 nm were achieved in PMMA and exhibited an exposure dose of 2000 pC/cm. To produce this pattern, the acceleration voltage that was used was 30 keV.^13^ The negative tone behavior of ZEP520A had also been investigated; Mohammad et al. demonstrated high-resolution patterns of 30 nm half-pitch lines while exhibiting an exposure dose of 5775 pC/cm at 10 KeV.^12^ Unfortunately, the dry etching selectivities of these resists are poor, where PMMA and ZEP520A exhibit a selectivity of 2:1 and 3:1, respectively.^14,15^ Moreover, Shipley advanced lithography (SAL601) is another negative tone e-beam polymer resist able to print a 40-nm-wide feature with a line exposure dose of 700 pC/cm at 30 KeV with a low etch selectivity equal to 5:1 of silicon to SAL601.^16^ Inorganic negative resists have emerged in the past few years. Hydrogen silsesquioxane is an inorganic negative resist and has shown an excellent resolution of 7 nm lines at 30 nm pitch obtained with an exposure dose of 7000 pC/cm at 30 KV.^17^ Different molecular negative resists have been published that show good performance.^18^ Tin-oxo cages are known to work as negative tone photoresists under EUV exposure and in EBL. An exposure dose of 1300 μC/cm^2^ is required to write 1 μm line/space patterns on the tin-oxo cage layer.^19^ A low molecular weight organic compound is reported as a negative tone molecular glass resist based on the bis-phenol A backbone. This material has a good contrast, well-resolved line pattern around 73.4 nm, and sensitivity of 52 μC/cm^2^ upon exposure in the EBL system.^20^ A new class of negative tone electron beam resist materials that exhibit high dry etch selectivity have been based on a family of heterometallic rings. In past studies, we have demonstrated negative tone resists that have high resolution (15 nm half-pitch) and ultrahigh etch selectivity for silicon (130:1 has been demonstrated) when subjected to a pseudo-Bosch inductively coupled plasma–reactive-ion etch (ICP–RIE).^21−23^

To design a new negative resist that is suitable for EBL applications, we have developed Monte Carlo simulations called Excalibur that track multiple generations of low energy cascading secondary electrons (SEs) to the carbon–carbon bond energy (3.6 eV) and it also tracks the generation of the Auger electrons (AEs).^23^ This is vital to understand the exposure mechanics which is used to determine the resolution and sensitivity of the resist. This can reduce the time to develop a new resist.^24,25^ The Excalibur simulation uses two models to describe the electron scattering behavior. The first utilizes the Joy model for electrons with kinetic energies above 500 eV.^26^ A second model for low energy was employed which exploits a quantum mechanical approach to electron scattering.^21,27^ The main electron interactions that are considered in this model are back-scattered electrons, SEs, and AEs. The molecular density, the effective atomic number, the average atomic weight, and the mean ionization potential play a fundamental role in simulating the interaction between the e-beam and the resist; moreover, the substrate type and the resist thickness have an important role for this model.^24^

The aim of this study is to identify new negative tone resists that can be used for photomask production suitable for EUVL. In this paper, two different resists have been tested to ascertain their highest possible resolution, highest possible sensitivity, and the etch selectivity characteristics. Preliminary Monte Carlo modeling led us to study a resist based on a metal–organic compound [NH_2_(CH_2_–CH=CH_2_)_2_][Cr_7_NiF_8_(Pivalate)_16_] **1** where seven chromium(III) centers and a nickel(II) form an octagon linked by fluorides and pivalate ligands.^21^ The exterior of the compound is entirely composed of tert-butyl groups, and this gives the compound high solubility in solvents suitable for preparing films on silicon substrates. The compound has a density of ρ = 1.212 g cm^–3^ with a large molecular weight (2192 Daltons). The beauty of this compound is that when spun on a surface, it forms a close-packed film.^22^ As we will show, this is a high-resolution but low-sensitivity resist. To improve the sensitivity, we hypothesized that the cadmium atomic number is larger than that of nickel, which will eject more SEs and start the cascade process as the primary electron (PE) traverses the resist film. To achieve this, the nickel atom was replaced by a cadmium atom to produce [NH_2_(CH_2_–CH=CH_2_)_2_][Cr_7_CdF_8_(Pivalate)_16_] **2**.

## 2 Experimental Procedure

In order to produce the resists as a liquid that can be spun on a silicon wafer, the resist molecules must be synthesized first, and the synthesis of resist **1** is given in ref (28). To synthesize the resist **2** compound, we mixed CrF_3_·4H_2_O (7.0 g, 38.7 mmol), pivalic acid (35 g, 342.7 mmol), and diallylamine (1.45 g, 14.9 mmol) in a Teflon flask and heated at 140 °C for 1.5 h with stirring, then CdCO_3_ (1.0 g, 5.8 mmol) was added and continuously stirred at 140 °C for 23 h. After this, the flask was cooled to room temperature and acetone (100 mL) was added and stirred for 1 h. The green product was filtered and washed with acetone (100 mL). Then the solid was stirred for 0.5 h in pentane (100 mL) and filtered and the solvent was removed on rotary. The residue was dissolved in toluene (600 mL) and filtered through a silica pad and the solvent was removed on rotary. The residue was washed with 50 mL acetone and dried, which resulted in a yield of 7.5 g.

To prepare 15 and 20 mg solutions, the resists **1** and **2,** respectively, are dissolved in 1 mL of t-butyl methyl ether. The films were deposited on a clean silicon substrate by a spin-coating technique and resulted in thicknesses of 24.5 and 31.2 nm. Resists **1** and **2** were exposed to an electron beam using a Sigma Zeiss scanning electron microscope. The electron beam was driven using a Raith Elphy plus 6 MHz pattern generator. The same pattern was exposed on both samples with single-pixel lines of 5 μm length separated by pitches of 100 down to 40 nm. The films were exposed using an acceleration voltage of 30 kV, where the current was 36 pA, and the step size was 2 nm. To achieve the highest possible resolution that gives repeatable results, a line dose is used. The base dose was 987 pC/cm and the dose factor was between **1** and 20. Both resists were developed in a bath of hexane for 10 s and blow-dried using dry N_2_. The lines were exposed to a dose ranging from 987 to 19,740 pC/cm with an increment of 0.1 pC/cm.

The dry etch experiments were carried out in an Oxford Instrument Plasma Pro 100 Cobra ICP etching system. The plasma is based on SF_6_/C_4_F_8_ (22 sccm:35 sccm) gases at a pressure of 10 mTorr with a deep RIE power of 20 W and a forward ICP power of 1200 W; the etching time was 40 s.

## 3 Results and Discussion

### 3.1 Monte Carlo Simulations

**Scheme 1:**
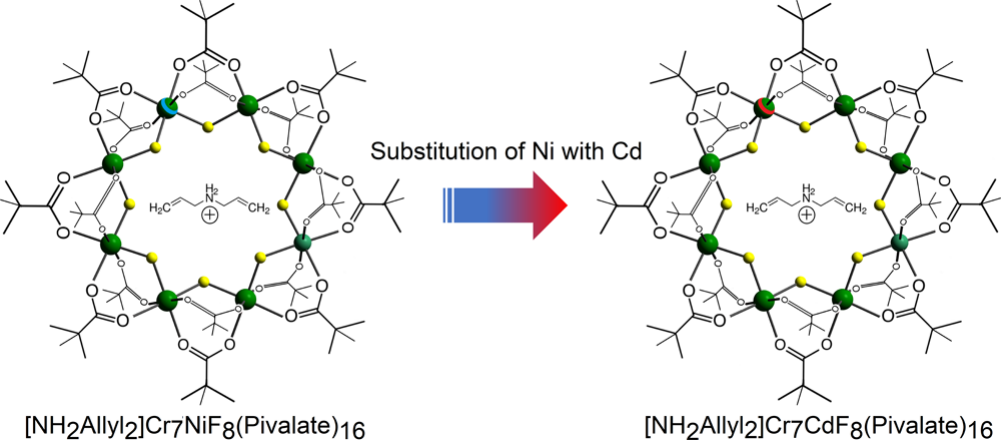
Structure of Resists **1** and **2**; Cr Green, Ni Green with a Blue Band, Cd Green with a Red Band, F Yellow, C Gray. H Atoms Omitted for Clarity

The Monte Carlo simulation called Excalibur was used to obtain a physical understanding of the electron scattering effects inside the **1** and **2** resist materials (Scheme 1). This is important, as these electrons are responsible for exposing the resist and then increasing the overall sensitivity. The uniqueness of our Excalibur Monte Carlo software is that the patterns can be inputted allowing the user to understand what the resolution of the resist should be while ascertaining what the exposure dose should be.

**Figure 1:**
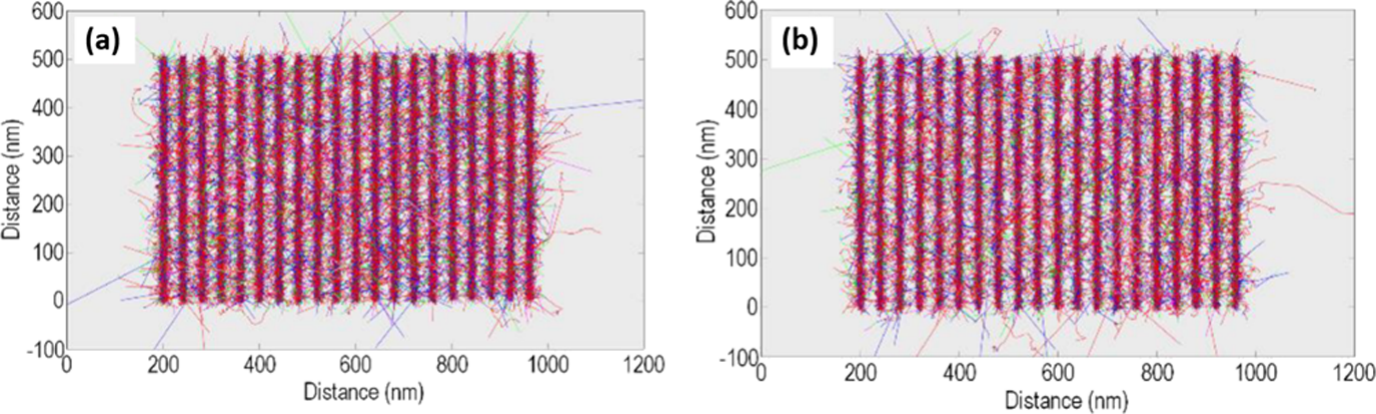
(a) Top-down view of the Monte Carlo simulation of the performance of **1** at 40 nm pitch on Si. (b) Top-down view of the Monte Carlo simulation of the performance of **2** at 40 nm pitch on Si.

Figure 1a shows the Monte Carlo simulation of the performance of **1** at 40 nm pitch. A pattern is able to be inputted into our Monte Carlo simulator which will be compared to the pattern obtained from the experimental. The inputted pattern consisted of 20 single pass lines (500 nm in length) that have a 40 nm pitch. The step size of the pattern is 2 nm giving 250 spots per line (pixel size). 500 incident PEs were used per spot giving a total of 2,500,000 incident PEs used for the entire pattern. These exposure parameters would be the exposure parameters that would be used to produce the lithography in the experimental.

The number of SEs and the number of AEs generated in the resist **1** were 313,454 and 179,025 electrons, respectively, and the incident electron energy was 30 keV. This is equivalent to 62.7 SEs and 35.7 AEs generated per spot. Comparing this to the simulation for **2** (Figure 1b), the Monte Carlo determined that the number of SEs that were generated inside the resist was 443,872 and 244,117 for the AEs, equivalent to 88.7 SEs and 48.8 AEs generated per spot.

It was found that the substitution of Ni with Cd increases the number of generated SEs and AEs by a factor of 1.4 and 1.37, respectively. Therefore, it predicts that the resist **2** will have an increased sensitivity when compared to resist **1**.

In the Monte Carlo simulations, the black lines represent the PEs, and the red lines are first order SEs generated from collisions with the resist during the writing with energies >500 eV. Cyan lines are second order SEs; purple lines are third order SEs; green lines are fourth order SEs; these SEs have associated energies <500 eV. The blue lines are back-scattered electrons. Five hundred 30 keV electrons were incident per spot and the step size was 2 nm; hence, 2.5 million electrons were used for each pattern.

From the simulations, it was found that **2** had a higher number of SE and AE generation in comparison to **1**. This is the effect of the cadmium atom because it had a higher atomic number (*Z*) and density (ρ) which led to increasing the scattering cross-section of the resist which inherently increases the number of available electrons that can be ejected from the shells of the cadmium atom in the resist. This will cause more inelastic collisions to occur, producing more SEs. This is advantageous because, as the atomic number of the atom increases more electrons are available in its outer shells that can be ejected out as SEs. These electrons have lower energy than that of the core electrons because they are further away from the nucleus. This means that the energy required to strike these out of the outer shells is less and the associated energy of the ejected SE is less too. These SEs will not have enough energy to traverse the resist laterally, thus, high resolution will be obtained. These electrons will do more damage in the immediate exposure area, by producing an electron cascade in the immediate exposure, thus lowering the required dose because each SE produced will lose more energy faster than its parent SE. The increase of SE collision events causes the solubility changes which renders the resist insoluble after the exposure process and is consequently left behind in the development process. A similar increase is seen in the generation of AEs. This is caused by the increased number of inelastic collisions at lower energies meaning a larger number of potential Auger emission events can occur.^29,30^

### 3.2 EBL

**Figure 2:**
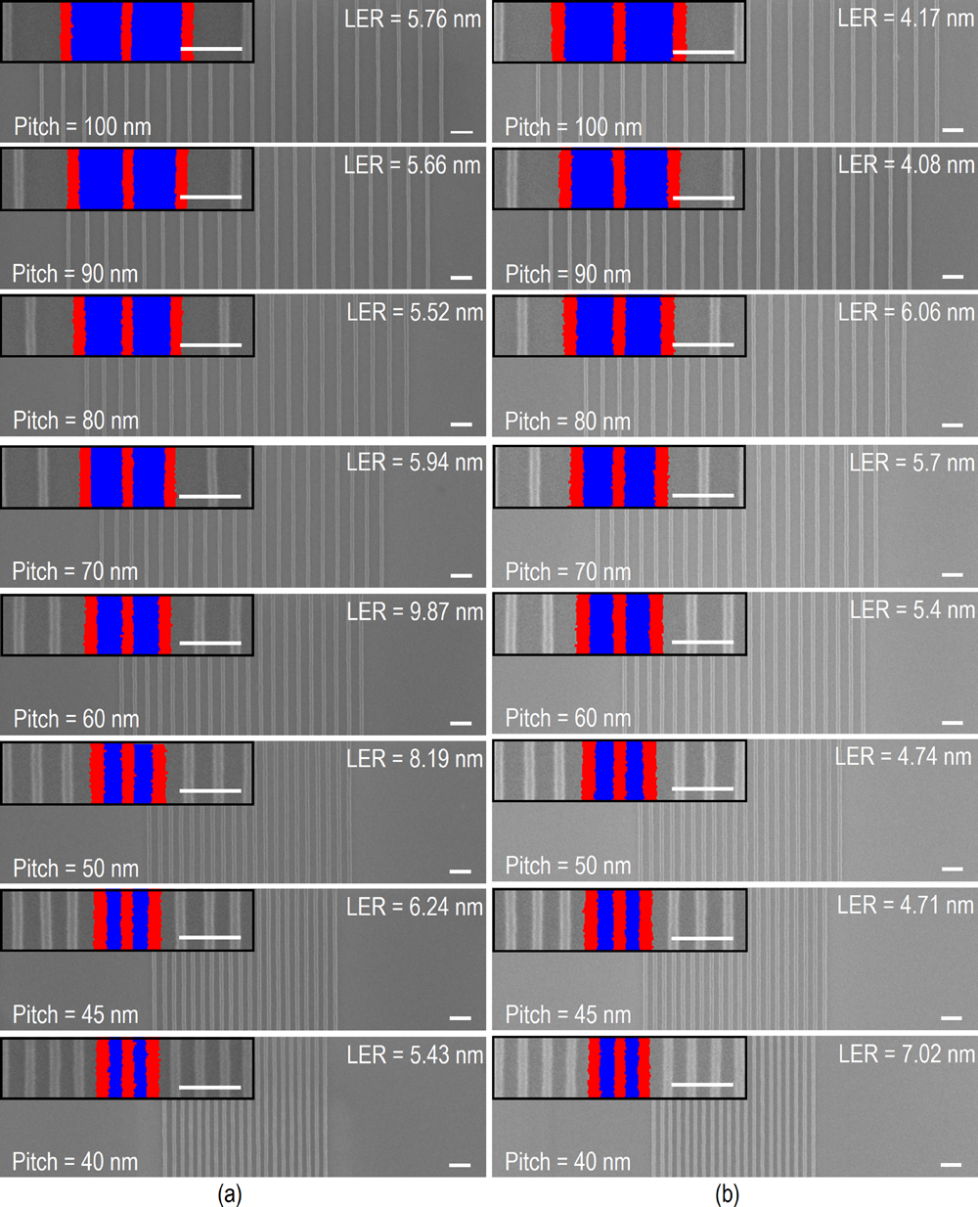
Top-down view of different pitch patterns fabricated in (a) [NH_2_Allyl_2_]Cr_7_NiF_8_(Pivalate)_16_, inset images of the LER measurements. (b) [NH_2_Allyl_2_]Cr_7_CdF_8_(Pivalate)_16_ resist on Si wafers. Inset images of the LER measurements. Scale bar is 100 nm and the inset scale bar is 100 nm. LERs were determined using 3σ.

Resists **1** and **2** were exposed using EBL. Figure 2a,b presents the nanostructures exposed at 11,351 and 7995 pC/cm for resists **1** and **2,** respectively. These patterns were obtained after a development process using hexane for 10 s. The patterns become nonsoluble after development due to a partial transformation to chromium oxide, and the solubility changes render the resist insoluble after the exposure process and is consequently left behind in the development process.^21^ It can be seen that all patterns were resolved, and the smallest resolved dense lines obtained for each resist were the 40 nm pitch as obtained in Monte Carlo simulations (Figure 1). A preliminary study with different exposure dose is done for both resists in order to obtain the optimum exposure dose. The optimum exposure dose results in straight continuous and uncollapsed lines. A smaller line edge roughness (LER) is obtained for resist **2** with a value equal to 4.17 and 7.02 nm for the 100 and 40 nm pitch, respectively.

**Figure 3:**
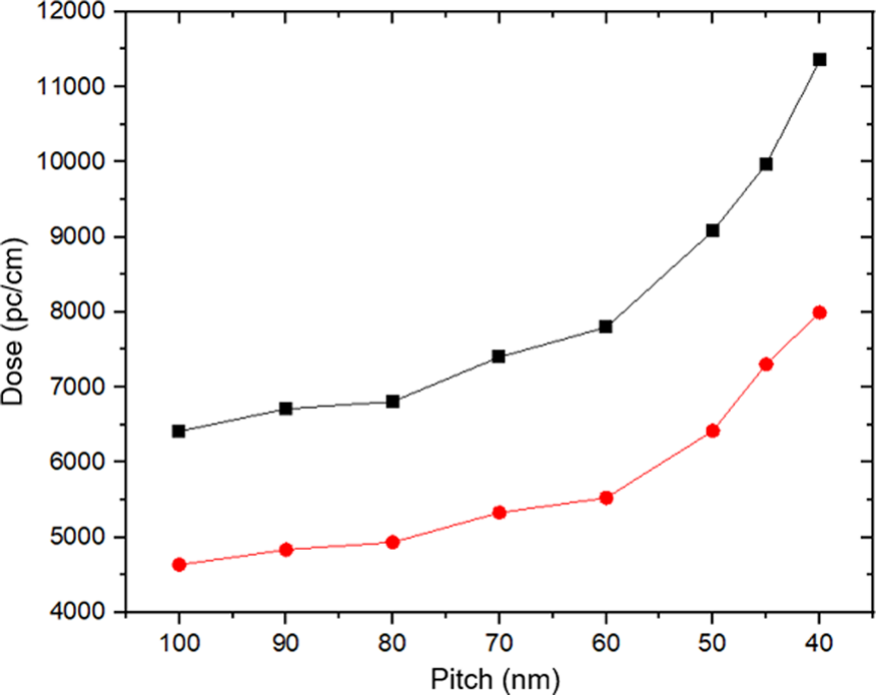
Exposure dose versus pitch for **1** (black squares) and **2** (red circles) on Si wafers.

The exposure dose for each pitch is presented in Figure 3, and it can be seen that resolved patterns were achieved across different doses depending on the pitch. It is evident the proximity effect plays a dominant role as the pitch of the pattern decreases, and the exposure dose required to produce each pattern increases from 6412 up to 11,351 pC/cm for 1; for **2,** the exposure dose increases from 4636 up to 7995 pC/cm. The sensitivity of **2** compared with **1** is improved by a factor of 1.4 simply by substituting the nickel atom with cadmium. The increased sensitivity illustrates a strong agreement between the simulation and the experimental results.

The exposure line doses as a function of the pattern pitch sizes for **1** and **2** deposits on silicon was examined. Figure 3 shows that the effective dose at 30 kV for both resists increases when the pitch increases especially when the pitch is smaller than 60 nm.

Upon the development stage of the process, capillary forces can play a fundamental role at smaller pitches, where the capillary forces from the solvent increase in between the lines and this force increases substantially as the pitch decreases. If the force is increased past the pattern aspect ratio threshold, then the pattern collapses. At a high pitch of 100 nm, the capillary force is not sufficient enough to cause bending and collapse because the exposed resist is stronger. However, as the pitch is reduced, the capillary force becomes stronger leading to the collapse of the patterns at the same exposure dose. This why a higher exposure doses is required to maintain completely straight lines.^31^ Producing an area pattern in micrometer size squares would require lower exposure doses due to the proximity effect between the point-to-point step size (which in our case was 2 nm), that would play a significant role in the exposure, leading to an increased sensitivity.

**Figure 4:**
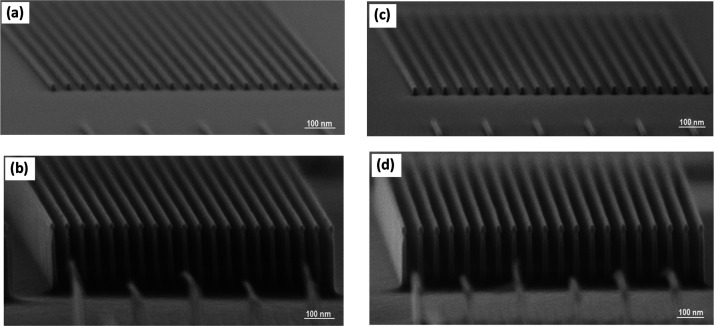
(a) Scanning electron microscopy (SEM) images of the 17 nm lines on a 40 nm pitch that was written at 30 keV in 1. (b) SEM of structure in panel (a) after a 40 s plasma etch. (c) SEM image of the 15 nm lines on a 40 nm pitch that was written at 30 keV in **2**. (d) SEM of the structure in panel (c) after a 40 s plasma etch.

Figure 4a,c shows lines that were exposed from thin films of **1** and **2** that were 24.5 and 31.2 nm thick, respectively. It can be seen that resists **1** and **2** produced high-resolution patterns of 17 and 15 nm, respectively. These patterns can be transferred to the silicon substrate using ICP–RIE using SF_6_ and C_4_F_8_ gases (Figure 4b,d).

This produces silicon nanostructures with a height of 214 nm. Both materials exhibit the same high etch selectivity to silicon in the presence of a SF_6_/C_4_F_8_ etch, allowing for the transfer of 20 nm wide lines into the substrates. It can be seen clearly that both resists remain on the top of the silicon structures. The effective etch rates by the SF_6_ plasma were determined to be 0.054 and 5.35 nm/s for the **1**, **2** materials and silicon, respectively. This result was repeated nine times and was determined that the silicon was etched approximately 99 times faster than the resist at the same etching conditions. This high selectivity is explained previously by the decomposition of both resists into chromium oxyflouride material, which is very stable with no chemical etch process between this material and the etch gases.^23^

## 4 Conclusions

In conclusion, it has been demonstrated that resists **1** and **2** are capable of producing sub-20 nanometer structures in silicon, spaced on a 40 nm pitch. This study identified a new chromium ring resist, which could improve the sensitivity of the resist whilst maintaining high resolution by replacing the nickel atoms with cadmium atoms for photomask application. Simulations and experimental studies suggest a potential improvement by a factor of 1.4 in exposure dose. The **2** material exhibits extremely high etch selectivity to silicon in the presence of an SF_6_/C_4_F_8_ etch, allowing for the transfer of 15 wide lines into the substrate with a selectivity of approximately 100:1 and a sensitivity of 7995 pC/cm for 40 nm pitch.
